# Variants in a Novel Epidermal Collagen Gene *(COL29A1)* Are Associated with Atopic Dermatitis

**DOI:** 10.1371/journal.pbio.0050242

**Published:** 2007-09-11

**Authors:** Cilla Söderhäll, Ingo Marenholz, Tamara Kerscher, Franz Rüschendorf, Jorge Esparza-Gordillo, Margitta Worm, Christoph Gruber, Gabriele Mayr, Mario Albrecht, Klaus Rohde, Herbert Schulz, Ulrich Wahn, Norbert Hubner, Young-Ae Lee

**Affiliations:** 1 Department of Pediatric Pneumology and Immunology, Charité Universitätsmedizin Berlin, Germany; 2 Max-Delbrück-Centrum for Molecular Medicine, Berlin, Germany; 3 Department of Dermatology and Allergology, Charité Universitätsmedizin Berlin, Germany; 4 Max Planck Institute for Informatics, Saarbrücken, Germany; University of Michigan, United States of America

## Abstract

Atopic dermatitis (AD) is a common chronic inflammatory skin disorder and a major manifestation of allergic disease. AD typically presents in early childhood often preceding the onset of an allergic airway disease, such as asthma or hay fever. We previously mapped a susceptibility locus for AD on Chromosome 3q21. To identify the underlying disease gene, we used a dense map of microsatellite markers and single nucleotide polymorphisms, and we detected association with AD. In concordance with the linkage results, we found a maternal transmission pattern. Furthermore, we demonstrated that the same families contribute to linkage and association. We replicated the association and the maternal effect in a large independent family cohort. A common haplotype showed strong association with AD (*p* = 0.000059). The associated region contained a single gene, *COL29A1,* which encodes a novel epidermal collagen. *COL29A1* shows a specific gene expression pattern with the highest transcript levels in skin, lung, and the gastrointestinal tract, which are the major sites of allergic disease manifestation. Lack of *COL29A1* expression in the outer epidermis of AD patients points to a role of collagen XXIX in epidermal integrity and function, the breakdown of which is a clinical hallmark of AD.

## Author Summary

Atopic dermatitis (AD, eczema) is a common chronic inflammatory skin disorder and a major manifestation of allergic disease. Typically, AD first occurs in early childhood, often preceding the onset of allergic airways disease, such as asthma and hay fever. A family history of allergic disorders is the single strongest predictor for AD, showing that genetic factors play a major role in the disease development. We have previously mapped a disease locus for AD on Chromosome 3q21, Now we have used a dense map of microsatellite markers and single nucleotide polymorphisms (SNPs) to find the underlying disease gene. We identified genetic markers in a subregion that showed association with AD, and replicated this finding in a large independent family cohort. The associated region contained a single gene, *COL29A1,* which encodes a novel collagen. We demonstrate that AD patients lack *COL29A1* expression in the outer epidermis, implicating collagen XXIX in epidermal integrity and function. The gene expression pattern of *COL29A1* in other organs, including the lung and the gut, suggests that this gene could have a role in a wider spectrum of allergic diseases and may provide a molecular link between AD and respiratory airways disease and food allergies.

## Abstract 3

The gene underlying atopic dermatitis susceptibility has been identified by gene mapping as expressing a novel collagen, whose expression is lacking in the outer epidermis of atopic dermatitis patients.

## Introduction

Atopic dermatitis (AD) is a chronic inflammatory skin disease that is characterized by intensely itchy skin lesions. AD is one of the most common chronic diseases in childhood affecting 10%–20% of children in industrialized societies [1], with a steady increase over the past decades [2,3]. Along with asthma and hay fever, AD is commonly associated with an abnormal immune response and the formation of allergy antibodies (IgE) against innocuous environmental allergens.

AD is often the first clinical manifestation of allergic disease. The onset of disease is typically observed during the first two years of life [4]. For the majority of affected children, AD heralds a lifetime of allergic disease. A susceptible child commonly passes a characteristic sequence of transient or persistent disease stages that is known as the “atopic march” which begins with AD and food allergy in the young infant and continues with the development of allergic airways disease later in childhood and adulthood [5]. The close familial and intra-individual association of the allergic disorder strongly suggests shared genetic etiology.

A strong genetic component in allergic disorders has been recognized almost a century ago. Cooke and van der Veer first reported that the relatives of patients are at significantly increased risk of developing allergic disease [6]. Even today, a positive family history for allergic disorders is the single strongest predictor for the development of AD [7]. Additional evidence for the importance of genetic factors in atopic disease comes from twin studies. The concordance rate for AD among monozygotic twins of about 80% far exceeds the concordance rate of 20% observed among dizygotic twins [8,9]. These data clearly indicate that the genetic contribution to the expression of AD is substantial. Furthermore, studies on the vertical transmission of AD and atopic disease show that children are more likely to inherit these disorders if the mother is affected (parent-of-origin effect) [10]. The predominance of maternal inheritance may be due to environmental factors such as uterine milieu or breast feeding, but they may also arise due to genetic mechanisms such as parent-specific gene expression (genomic imprinting) [11].

AD and atopic disorders are regarded as multifactorial conditions, the onset and severity of which are influenced by both genetic and environmental factors. The data are consistent with an immune etiology shared by all allergic diseases and a congenital target organ defect, the penetrance of which is modified by multiple environmental factors during early childhood. The identification of genes underlying AD and allergic disorders has the capacity to define primary physiologic mechanisms, thereby clarifying disease pathogenesis, identifying pathways and targets for therapeutic intervention.

Although several genome-wide linkage screens for AD have been conducted [12–14], there was no substantial overlap between the identified regions of highest linkage, and the underlying genes remained elusive [15]. We have previously mapped a major susceptibility locus for AD on Chromosome 3q21 [16]. Here we report the identification and characterization of a novel epidermal collagen gene as the underlying disease gene.

## Results

**Figure 1:**
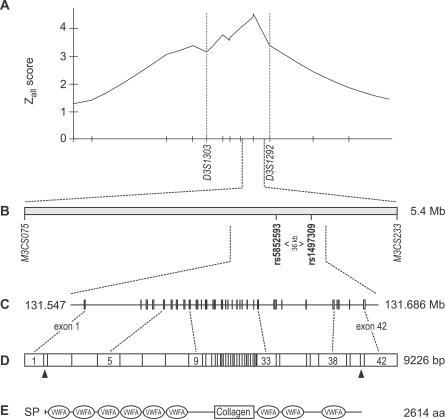
Positional Cloning Strategy for the AD Disease Gene on Chromosome 3q21 (A) The candidate region spanned 12.75 cM between markers *D3S1303* and *D3S1292*. The *y*-axis depicts the *GENEHUNTER* nonparametric Z_all_ as previously reported [16]. (B) Fine mapping with 96 microsatellite markers narrowed the interval to 5.4 Mb between markers *M3CS075* and *M3CS233*. An association scan using 212 SNPs of the region revealed association of AD with two adjacent SNPs, rs5852593 and rs1497309. Genotyping of 16 additional SNPs refined the associated region. (C) Genomic positions of the 42 exons of *COL29A1* are shown. The gene entirely encompasses the associated region. (D) The *COL29A1* mRNA consists of 9226 bp. Translation start site and stop codon are indicated. (E) The predicted open reading frame encodes a protein of 2614 amino acids including a secretion peptide (SP), six N-terminal and three C-terminal vWAs, flanking a short collagen triple helix.

To narrow the candidate region spanning 12.75 centiMorgans (cM) (13.5 Mb) on Chromosome 3q21 (Figure 1A), 96 additional microsatellite markers at an average distance of 140 kb were genotyped in 199 affected sibling families with AD from the original linkage scan. Linkage analysis yielded a 1-lod support interval of 5.4 Mb between the markers M3CS075 and M3CS233 (Figure 1B). Subsequently, we conducted an association scan of the 5.4-Mb region using 212 single nucleotide polymorphisms (SNPs) at an average distance of 25.5 kb (Table S1). The SNPs were selected primarily to cover known and predicted genes. Because we had observed a strong maternal effect in the linkage study [16], we chose a family-based association analysis that allowed us to search for risk alleles preferentially transmitted from the mother [17]. Two adjacent SNPs, rs5852593 (*p* = 0.0079) and rs1497309 (*p* = 0.016), located 36 kb apart, were associated with AD (Table S1 and Figure 1B). To define the critical region, we typed 16 additional SNPs. Eight of these markers showed association with AD and a maternal transmission pattern to affected children, which was consistent with our previous linkage results (Table 1). The strongest association with a single marker was observed for rs4688761 (*p*_all_ = 0.0016, *p*_maternal_ = 0.0006). We selected eight markers spanning 96 kb that were associated with AD and carried nonredundant information based on the linkage disequilibrium (LD) in the region, and we performed haplotype analysis which confirmed the results (transmissions (T)_all_:non-transmissions (NT)_all_ = 222:168, *p*_all_ = 0.0076, T_mat_:NT_mat_ = 105:68, *p*_maternal_ = 0.0070). In addition, we assessed the significance of the difference in maternal-versus-paternal haplotype over transmissions empirically using the permutation procedure for parent-of-origin transmission disequilibrium test (TDT) implemented in PLINK (P_POO_maternal emp_ = 0.014) [18].

**Table 1:**
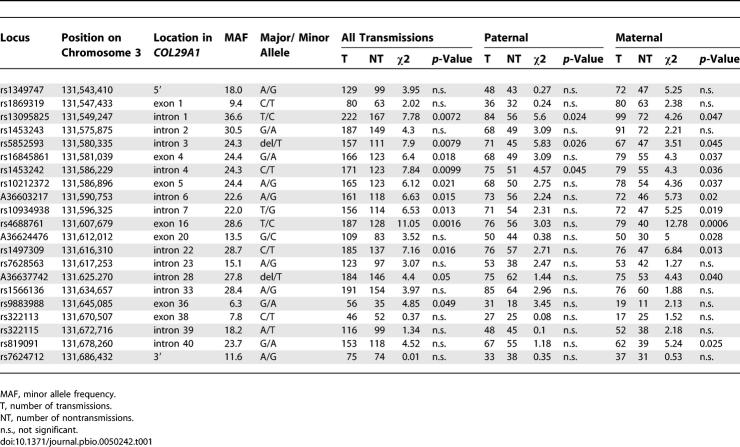
Results of the ASPEX sib_TDT for SNPs in the Region of *COL29A1* for AD in the Discovery Cohort

Next, we investigated whether the observed association accounted for the linkage in the region. We used the marker that had shown the strongest association, rs4688761, to identify 73 of the 199 families from the original linkage scan in whom the disease-associated allele had been transmitted to affected offspring. Nonparametric linkage analysis in the 73 associated families yielded significant evidence for linkage (*Z*_all_ = 4.18 versus 4.31 in the complete cohort), demonstrating that the majority of the linkage signal was attributable to these families. The significance of this finding was assessed by performing nonparametric linkage analysis in 10,000 random selections of 73 families. An empirical significance level was calculated as the proportion of replicates for which the maximum *Z*_all_ score was equal or greater than that obtained in the actual analysis. The probability of obtaining a *Z*_all_ score of ≥4.18 by chance in a random selection of 73 families was estimated by 10,000 simulations to be 0.027.

To confirm the association result, we used a large independent replication set consisting of 292 complete nuclear families including 481 children with AD. We genotyped the selected eight SNPs covering an interval of 96 kb that were associated with AD in the discovery dataset. We confirmed the association with AD across all markers with the strongest association with AD observed for marker A36637742 (*p* = 0.00038), which also showed a significant overtransmission of the maternal allele (*p* = 0.0013) (Table 2). The association remained significant after correction for multiple testing. For each marker, it was the more common allele that was overtransmitted. Haplotypes were constructed over the region, which confirmed the association with AD and showed that this phenotype is associated with the overtransmission of the most common haplotype (haplotype frequency 65%, Table 3), and of the maternal allele (*p*_maternal_ = 0.000025 for AD, P_POO_maternal emp_ = 0.033). Next, we compared the AD status among the parents: in the discovery cohort, significantly more mothers (*n* = 63) than fathers (*n* = 19) suffered from AD (odds ratio [OR] 4.39, 95% confidence interval [CI] 2.51 – 7.68, *p* = 5.46 × 10^−8^). Similarly, in the replication cohort, mothers (*n* = 83) were significantly more frequently affected with AD than fathers (*n* = 55) (OR 1.71, 95% CI 1.16 – 2.52, *p* = 8.3 × 10^−3^). To assess whether the parent-of-origin effect that was observed originated from the discrepancy in AD prevalence among the parents, we compared haplotype transmissions from affected and unaffected mothers. In the discovery cohort, the excess in maternal transmissions was not predominantly attributable to affected mothers (T:UT = 24:21, *p* = 0.14) compared to unaffected ones (T:UT = 81:47, *p* = 0.0059). Similarly, affected mothers (T:UT = 25:17, *p* = 0.14) of the replication cohort did not contribute a larger transmission excess than the unaffected ones (T:UT = 70:29, *p* = 0.00013). We conclude that the observed maternal overtransmission pattern in both cohorts was not due to the higher AD prevalence among mothers.

**Table 2:**
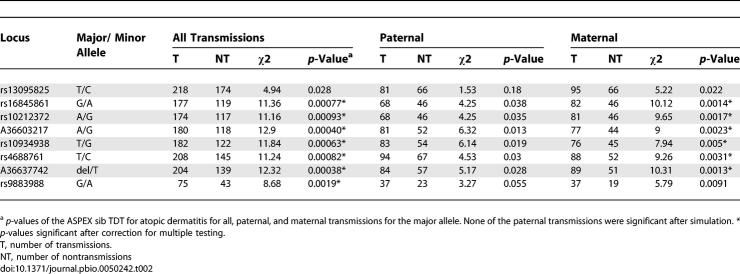
Association Analysis of Single Markers for Atopic Dermatitis in the Replication Dataset

**Table 3:**
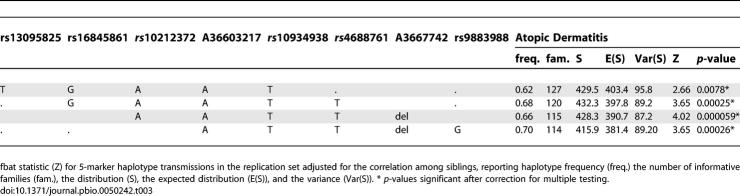
Haplotype Analysis for AD in the Replication Dataset

**Figure 2:**
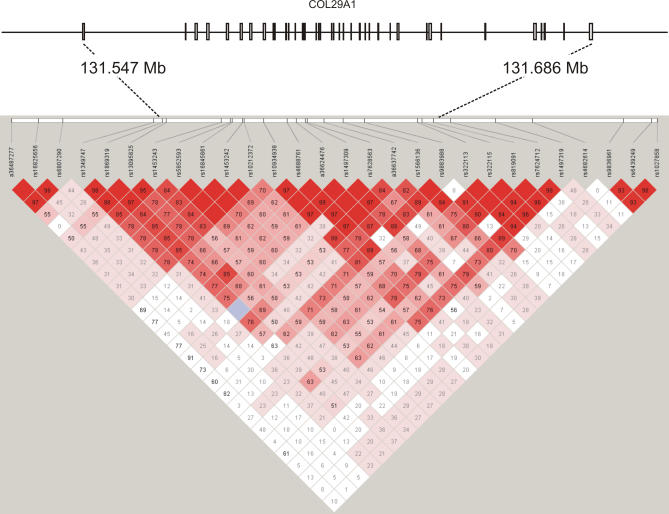
Pairwise LD Values (D′) Between 28 SNPs Based on Genotypes of the Founders in the Discovery Cohort Boxes contain the LD values (D′) between the respective markers indicated on top. Higher LD values correspond to a darker shade of red. Positions on Chromosome 3 are given in Mb; 131.547 denotes the start and 131.686 the end of *COL29A1* on the genomic sequence. Boxes on the horizontal bar represent the 42 exons of *COL29A1*.

A database search within the associated 96-kb interval revealed a single predicted gene, *FLJ35880*, extending 11.6 kb into the associated region. No other expressed sequence tag was detected. To identify any additional transcripts, we used putative exons predicted with The National Center for Biotechnology Information (NCBI) Modelmaker within and bordering the critical region to perform rapid amplification of cDNA ends (RACE) from human skin mRNA. We thus identified a single transcript of 9226 bp that consisted of 42 exons (Figure 1D) and included all eight exons of *FLJ35880*. The corresponding gene spanned 139 kb of genomic sequence and completely encompassed the associated region. Pairwise LD measures (D′) of the genotyped markers indicated that the gene was contained within one 170-kb region of increased LD (Figure 2), whereas the neighboring genes, *LOC440978* and *LOC131873,* were located in separate blocks. The LD structure was consistent with the data for the European population in the HapMap database (Figure S1) [19]. The size of the transcript was confirmed by Northern blot hybridization with an *FLJ35880*-specific probe detecting a single transcript of 9.6 kb in human skin mRNA (unpublished data), which was in good agreement with the RACE experiments.

The open reading frame yielded a protein of 2614 amino acids with an estimated molecular weight of 289.9 kDa. The predicted protein contained a collagenous domain in the central part and was therefore classified as a new member of the collagen superfamily, collagen XXIX. A BLAST search revealed the human collagen VI alpha 3 chain as its closest neighbor (32% identity). Homology with collagen VI alpha 3 was further strengthened by a similar domain architecture consisting of six N-terminal and three C-terminal von Willebrand factor–type A domains (vWAs) flanking a short collagen triple helix (Figure 1E), and an 18–amino acid secretion signal [20]. Sequence analysis of all 42 exons in 46 unrelated children with AD and 2 unaffected individuals revealed 13 common and six rare sequence variations (frequency <2%) predicted to cause nonsynonymous amino acid substitutions (Table S2). Four variants were located within the triple helix repeat of a collagen domain, however none of them changed the first glycine residue in the repeating sequence patterns (Gly-X-Y), which is critical for triple helix formation [21]. In addition, we performed in silico comparisons of the six variants located within a vWA with the crystal structure of the human vWF A3 domain. One variant, V669G, affects an amino acid within a highly conserved stretch of eight amino acids. The mutation of an adjacent amino acid within this conserved region of a vWA in the homologous gene *COLVIA1* has been reported to cause the monogenic muscle disorder Bethlem myopathy [22]. This variant, however was rare (allele frequency 1,3%). One additional variant (E455K) is located in a region predicted to be important for integrin–collagen interaction, and another one (M56T) is located in a helix near a rather conserved region that may also affect protein–protein interaction. All coding SNPs were genotyped in the discovery cohort. Four of them showed a positive association with AD that did, however, not account for the observed haplotype association (Table S2).

**Figure 3:**
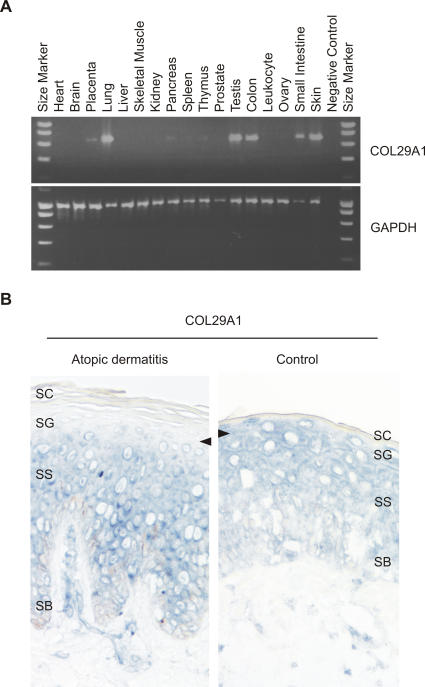
Gene Expression Analysis of *COL29A1* (A) RT-PCR analysis of collagen XXIX in human tissues. (B) In situ gene expression analysis of *COL29A1* in AD skin. In situ hybridization results of a *COL29A1*-specific antisense probe on cryostat sections (5 μm thick) of an AD skin biopsy (left) and a normal human control (right) are shown. *COL29A1* mRNA detected by the digoxigenin-labelled probe is stained in blue (BCIP/NBT staining). The arrows point the different gene expression in the upper spinous and granular layer of the epidermis of an AD patient and a normal human control. Stratum basale (SB), stratum spinosum (SS), stratum granulosum (SG), and stratum corneum (SC) are indicated.

Gene expression analysis in human tissues revealed a tissue-specific expression pattern of *COL29A1*. The highest expression was observed in the skin, but also in the lung, small intestine, colon, and testis (Figure 3A). Overall, *COL29A1* expression is moderately low compared to more abundant epidermal transcripts such as keratin 10 (unpublished data).

To specify the expression sites in the layers of the skin and to assess the role of collagen XXIX in AD, we performed in situ hybridization using a *COL29A1*-specific cRNA-probe on skin biopsies of five patients with AD and five healthy patients (controls). In normal skin, *COL29A1* was exclusively expressed in the epidermis with the strongest staining in the suprabasal viable layers. In contrast, the skin of patients with AD revealed a striking absence of *COL29A1* mRNA staining in the most differentiated upper spinous and granular layers (Figure 3B and Figure S2). No significant difference in the expression of *COL29A1* was observed comparing patients with AD to controls (1.28- ± 0.53-fold down-regulation in AD patients, *p* = 0.41) using quantitative Taqman reverse-transcriptase (RT)-PCR. These results indicate that while differences in mRNA quantity were not detected, AD patients show a distinct abnormal cellular distribution pattern of *COL29A1* expression in the differentiated outer epidermis.

**Figure 4:**
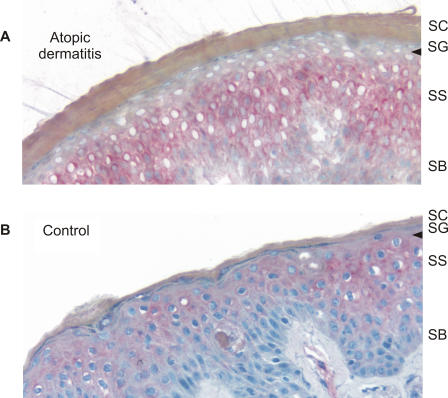
Immunohistochemical Analysis of Collagen XXIX Expression in AD Skin Cryostat sections (5μm thick) of an AD skin biopsy (A) and a normal human control (B) are shown. In normal, human skin, collagen XXIX is expressed in the epidermis. In patients with atopic dermatitis, a striking lack of collagen XXIX staining was observed in the viable outermost spinous and granular layers of the epidermis (arrow). Stratum basale (SB), stratum spinosum (SS), stratum granulosum (SG), and stratum corneum (SC) are indicated. Collagen XXIX was stained with fuchsin (red). Sections were counterstained with hematoxylin (blue).

We generated a polyclonal antibody to visualize the collagen XXIX protein in the skin of five patients with AD and five normal controls, including four and three of each group, respectively, in whom in situ analysis was performed. Consistent with the in situ findings, we observed collagen XXIX staining in the differentiated suprabasal layers of the epidermis in normal human skin and a remarkable absence of staining in the most differentiated upper spinous and granular layers (Figure 4 and Figure S3).

## Discussion

In a whole-genome linkage scan for AD, we previously identified a susceptibility locus on human Chromosome 3q21. The candidacy of this chromosomal region was further supported by Bradley et al., who mapped a locus for AD severity in close proximity (3q14) in a Swedish population [13]. By positional cloning, we have now identified the disease-causing gene, *COL29A1,* which encodes a novel epidermal collagen.

The disease gene was located in a two-staged investigation consisting of systematic linkage and association scanning of the region and subsequent confirmation of the association in a large, independent, replication dataset. In the first stage, we genotyped additional microsatellite markers in the candidate region, which narrowed the initial 12.75-cM linkage interval to 5.4 Mb. The association analysis with an average marker distance of 25.5 kb, finally, revealed an association that was confined to a haplotype block of 170 kb, which included a single gene, *COL29A1*. Pairwise LD measures indicated LD across the entire gene and defined two subblocks of increased LD. The strongest association was observed within the 96-kb subblock encoding the collagenous domain and the C terminus of collagen XXIX. A rapid decay of LD at the borders of the *COL29A1* haplotype block and lack of association of the SNPs located within the neighboring genes clearly limited the association to *COL29A1*. In addition, we demonstrated in the discovery dataset that the families that contributed to the association of SNP rs4688761 with AD also accounted for most of the linkage signal. This finding corroborated that variants in *COL29A1* explained the previously reported linkage of AD to 3q21 [16]. Finally, we confirmed association in a large independent family cohort, making *COL29A1* the first AD susceptibility gene, to our knowledge, that is identified by positional cloning.

Consistent with the linkage analysis, we found a maternal transmission pattern to affected offspring in both family cohorts. Although the sexes were equally represented among the affected children of both cohorts, we observed a marked maternal preponderance in AD status among the parents in both cohorts. This finding clearly supports the notion of a maternal effect. It may, however, also raise the question whether the maternal overtransmission pattern observed for the *COL29A1* haplotype was due to the different prevalence of AD in mothers and fathers. This is unlikely to be the case, because the analytical tools used in this study, nonparametric linkage and TDT, do not take into account the parental phenotype. Furthermore, we showed that the observed maternal effect was not predominantly attributable to transmissions from affected rather than unaffected mothers. Although parent-of-origin effects have not previously been reported for genes from Chromosome 3q21, they have been observed at other loci influencing allergic disease [23]. Tissue-specific imprinting of genes encoding extracellular matrix (ECM) proteins has been reported in the mouse [24], and their disruption has been shown to impair skin structure and function [25]. Interestingly, *COL29A1* is expressed in human placenta, an organ of embryonic origin. Apart from classic genomic imprinting mechanisms, maternal effects may be due to an interaction of the child's genotype with the maternal environment during prenatal life.

Sequencing of the exons and promoter region revealed 19 nonsynonymous coding SNPs, six of which were located within a vWA. In silico analysis of these variants revealed only one rare mutation altering a highly conserved amino acid, but all of them may affect protein–protein interaction. None of the nonsynonymous coding SNPs explained the observed association on its own. It has been demonstrated in other complex diseases that multiple independent variants may occur in a single disease gene [26]. It is therefore possible that several variants or combinations thereof which are associated with the most common haplotype of *COL29A1* are involved in the disease pathogenesis. The functional influence of the associated variants remains to be determined.

Involvement of *COL29A1* in AD is further supported by its tissue- and cell-specific expression pattern. Like *COL29A1*, a growing number of collagens recently identified show a restricted expression pattern. These are not mainly found in fibrous connective tissue, but rather in the ECM of more specialized tissue structures pointing to a distinctive function of these proteins [27]. Highest *COL29A1* expression was observed in the skin, but also in other epithelial tissues such as the lung, small intestine, and colon, which are the main manifestation sites of allergic disorders, including asthma and food allergies. This gene expression pattern might indicate a role of collagen XXIX in a wider spectrum of allergic diseases and suggests a molecular link between AD, respiratory airways disease, and food allergies, which are epidemiologically closely associated [28,29].

In human skin, collagen XXIX was detected throughout the viable layers of the epidermis with an increase toward the differentiated cells of the granular layer. Comparative expression analysis of *COL29A1* by in situ hybridization and immunohistochemistry in skin biopsies of patients with AD and healthy controls revealed a distinct lack of *COL29A1* mRNA and protein in the outer viable layers of the epidermis. The process of epidermal stratification is tightly regulated by an increasing gradient of extracellular Ca^2+^ concentration and a specific special and temporal expression pattern of transcriptional regulators [30]. Our findings indicate that the specific cellular milieu acquired during terminal epidermal differentiation affect the regulation or degradation of *COL29A1* mRNA in the outer epidermis. However, our findings do not allow us to distinguish between these two processes.

Lack of collagen XXIX in the outer epidermis of AD patients indicates that a defective ECM may give rise to the disease, proposing a new pathomechanism for AD. Collagens are the most abundant ECM proteins in vertebrates and play a crucial role in maintaining tissue integrity. Their importance for tissue function has been highlighted by the wide spectrum of human diseases caused by mutations in collagen genes [31]. Although a large number of collagens in the connective tissue–rich dermis have been characterized, little is known about collagens in the ECM of the epidermis [32,33]. Collagen XXIX belongs to the vWA containing collagens. They form filaments with globular domains containing the vWA motifs, which are involved in protein–ligand interactions for the organization of tissue architecture and cell adhesion [34]. It is therefore conceivable that collagen XXIX plays an important role in keratinocyte cohesion. Lack of collagen XXIX may facilitate antigen penetration through the skin, which may explain the association found between *COL29A* variants and allergic sensitization, a common feature in AD patients [35]. Recent findings indicate that structural and functional integrity of the epidermis is a key factor in the development of AD [36] and in the disease progression to allergic airways disease [37].

Furthermore, ECM collagens influence the migration of epidermal antigen-presenting Langerhans cells and T cells [38,39] and may thus play an important role in the initiation and maintenance of cutaneous immune responses. In addition, ECM collagens participate in immune regulation by binding to inhibitory immune receptors [40], rendering collagen XXIX an interesting novel susceptibility gene for AD. Impairment of the immune defense function of the skin is a clinical hallmark of AD. Patients with AD show a diminished resistance against microbes resulting in the colonization of nonlesional skin with Staphylococcus aureus in nearly 90% of patients and an increased susceptibility to bacterial and viral skin infections [41]. Our findings led to the identification of collagen XXIX as a novel component of the epidermal ECM and propose a new disease mechanism in the etiology of atopic dermatitis and allergies.

## Materials and Methods

### Human subjects.

The diagnosis of AD was made according to standard criteria, as previously described [16]. Recruitment was restricted to patients with an age of onset below 2 y and moderate to severe disease expression. Total IgE levels and levels of specific IgE against 12 common environmental allergens were determined using ImmunoCAP (Phadia AB; http://www.phadia.com/). Allergic sensitization was defined as either the presence of specific IgE to at least one allergen (detection limit 0.35 kU/l) or a total serum IgE level elevated above the age-specific norm. The institutional review boards of the participating centers approved the study protocol and informed consent was obtained from all probands or their legal guardians.

One discovery study sample and one replication sample were investigated. All families were of European origin. The discovery data set consisted of 199 complete affected sibling families composed of 427 children with AD that were studied in the original genome scan. [16] The replication set consisted of 292 families including 481 children with AD with an age of onset ≤2 y and moderate to severe disease expression. Among AD patients in the discovery and the replication cohorts, the proportion of boys was 52% and 50.9%, and the proportion of children with allergic sensitization was 74% and 72.1%, respectively.

Punch biopsies of human skin were obtained from six patients with AD and 7 healthy donors aged 24–55 y with written informed consent. Specimens were prepared for in situ hybridization and immunohistochemistry as described below.

### Microsatellite genotyping.

In the first stage, fine mapping with microsatellite markers was performed in the discovery dataset. 96 short tandem repeat markers were selected within the interval between *D3S1303* (126.07 cM) and *D3S1292* (138.82 cM) from the Genethon (http://www.genethon.fr/) and Marshfield (http://research.marshfieldclinic.org/genetics/) databases, or were identified by screening human genome sequence data for short tandem repeats. Fluorescence-based semi-automated genotyping was performed as previously described [16]. Primer sequences, amplification conditions, and allele size are available on request.

### Association scan with SNPs.

We performed an association scan of the 5.4-Mb region using 212 SNPs at an average distance of 25.5 kb. The SNPs were selected from the NCBI database (http://www.ncbi.nlm.nih.gov/projects/SNP/) to cover known and predicted genes. To determine the allele frequency of the polymorphisms, we amplified 600 to 800 bp surrounding each SNP by PCR for resequencing in 31 unrelated Caucasian individuals. Markers with a minor allele frequency (MAF) of >5% were selected for genotyping. To identify functional variants in collagen XXIX, we sequenced all 42 exons including the exon–intron boundaries and 5.1 kb of the promoter region in 46 unrelated patients with AD and two controls. Sequencing was performed on an ABI3730 DNA sequencer (Applied Biosystems; http://www.appliedbiosystems.com) using standard procedures.

We carried out SNP genotyping using TaqMan allelic discrimination, with probes and primers designed and synthesized by the supplier (Applied Biosystems), or by pyrosequencing using PSQ HS 96A (Pyrosequencing AB; http://www.biotagebio.com/). The averages genotyping success rate was 97.9%. All primer and probe sequences are available on request.

### Statistical analysis.

We performed linkage analysis of the microsatellite data using Genehunter V. 2.1 [42]. Each SNP was checked for compliance with Hardy-Weinberg equilibrium (HWE) in the parent population using a χ^2^ test, and those markers that were not in HWE were excluded from the analysis. We calculated pairwise LD between each marker pair using the D′ statistic.

In view of the strong imprinting effect at our locus, we conducted family-based association tests, because this strategy allows us to determine the parental origin of an associated allele. In the affected sibling families, we used the sib_TDT of the ASPEX software that performs a permutation procedure to calculate empirical *p-*values that are independent of linkage within families [17]. Furthermore, to assess the significance of the maternal effect, we calculated empirical *p-*values for the difference in maternal versus paternal haplotype transmissions using the parent-of-origin TDT implemented in PLINK [18].

The sib_TDT was also used in the analysis of eight markers in the replication dataset. The significance level of the replication results was assessed empirically. Using all pedigrees and all genetic markers used in the actual analysis, we generated 10,000 replicates using Allegro V1.2c [43] and conducted an association analysis as in the original dataset. An empirical significance level was calculated as the proportion of replicates for which the maximum χ^2^ score was greater than that obtained in the real dataset. All *p-*values are two-sided, significance was defined as statistical evidence expected to occur 0.05 times at random in the analysis. For multipoint analysis, we used the FBAT tools package [44] to generate haplotypes and performed family-based association tests for five marker haplotypes using the empirical variance option to adjust for correlation among sibling genotypes. To evaluate parent-of-origin effects in the multipoint analysis, we estimated haplotypes using MERLIN, recoded the haplotypes as alleles, and performed the sib_TDT using ASPEX.

### In silico protein analysis.

Protein sequences were retrieved from the UniProt (http://www.uniprot.org) and Ensembl (http://www.ensembl.org) databases. The domain architecture of the collagen XXIX protein was retrieved from the NCBI conserved domain search website (http://www.ncbi.nlm.nih.gov:80/Structure/cdd/wrpsb.cgi). The following domains were found in collagen XXIX and analyzed further: cd01472 (vWA_collagen), cd01450 (vWFA_subfamily_ECM), cd01470 (vWA_complement_factors), cd01465 (vWA_subgroup), and Pfam01391 (collagen triple helix repeat). To predict the 3D structure of the vWF protein domains in collagen XXIX, we explored the structure prediction results returned by the web servers GenTHREADER (http://bioinf.cs.ucl.ac.uk/psipred/) and FFAS03 (http://ffas.ljcrf.edu). Based on their very similar predictions, the human vWF A3 domain was chosen as the structural template to analyze the position of *COL29A1* SNPs in 3D and to predict their potential effect on protein function.

### Exon identification and characterization of the collagen XXIX gene.

A 2447-bp sequence from a human testis cDNA library which covered eight exons was the starting point for the characterization of *COL29A1*. Using rapid amplification of cDNA ends together with the Model Maker of NCBI, we identified a total of 42 exons and determined the transcription start site as well as the 3′ end of the *COL29A1* transcript in cDNA from human skin. The complete sequence of the transcript was confirmed by cloning and sequencing of the full-length cDNA. To explore the potential gene function of *COL29A1*, the protein sequence was predicted (http://us.expasy.org/tools/dna.html), a domain search was performed (http://www.sanger.ac.uk/Software/Pfam/search.shtml) [45], and the presence and location of signalling peptides was analyzed (http://www.cbs.dtu.dk/services/SignalP/) [46].

### Gene expression analysis.

We examined tissue-specific expression of *COL29A1* using oligo(dT)-primed cDNA of 17 different human tissues. cDNA samples of 16 tissues were from the human MTC Panels I and II, which are standardized for the expression of GAPDH (BD Biosciences; http://www.bdbiosciences.com). In addition, human skin poly(A)+ RNA (BD Biosciences) was transcribed into cDNA using the Transcriptor First Strand cDNA Synthesis Kit (Roche Diagnostics; http://www.roche.com). PCR was performed using *COL29A1*-specific primers 5′-GTTCTAACCAGAATGTATAATCATC (forward) and 5′-TAATTCCCAAGAACATCTCTGGT (reverse), yielding a product of 694 bp, and the GAPDH control primers supplied with the MTC panels.

For in situ hybridization, we generated a plasmid by cloning a *COL29A1*-specific PCR product amplified from human skin cDNA [5′-ACCTTAGGAGACAGGGTTGCT (forward); 5′-AGTTCCAATCTGGCTTGTGG (reverse)] into the pCRII vector (Invitrogen; http://www.invitrogen.com). We synthesized antisense and sense digoxigenin-labeled riboprobes using the Dig RNA Labeling Kit (Roche Diagnostics). Punch biopsies of human skin were obtained from five AD patients and five healthy donors, immediately fixed in 4% paraformaldehyde for 4 h, cryoprotected in 30% sucrose overnight, and embedded in Tissue-Tek (Sakura; http://www.sakura.com) for cryosectioning. 10 μm cryosections were mounted on slides and dried for 15 min at 50 °C. Sections were postfixed in 4% paraformaldehyde for 7 min at 4 °C and acetylated with 0.25% acetic acid for 10 min. Sections were prehybridized for 3 h and hybridized overnight at 50 °C with digoxigenin-labeled riboprobes. After hybridization, sections were washed twice with 2 × SSC at 53 °C and once with 0.1 × SSC at 58 °C. For detection of the hybridized probe, slides were incubated with BCIP/NBT staining solution (Roche Diagnostics) for 4 d according to the manufacturer's recommendations.

To quantify gene expression in skin specimens, total RNA was isolated from 160 μm cryosections of skin biopsies using the RNeasy Mini Kit (Qiagen; http://www.qiagen.com). RNA was reverse transcribed into cDNA with random hexamer primers using the Transcriptor First Strand cDNA Synthesis Kit (Roche Diagnostics). Taqman real-time PCR was performed with iTaq SYBR Green (BioRad; http://www.biorad.com) and gene-specific PCR products were detected on the ABI PRISM 7900 sequence detection system (Applied Biosystems). All measurements were performed in duplicate. *COL29A1* expression was normalized for 18S rRNA expression. Differences in gene expression were calculated using the ΔΔct method and were expressed as fold change. Gene-specific primers were as follows; *COL29A1*-forward, 5′-CCACCCTCTGGATCATCACT, *COL29A1*-reverse, 5′-GTTTTCTGTGCCACCGTTCT, KRT10-forward, 5′-CTGAAACCGAGTGCCAGAAT, KRT10-reverse, 5′-GCCTCCGGAACTTCCCTCT, 18S rRNA-forward, 5′-GGATGCGTGCATTTATCAGA, 18S rRNA-reverse, 5′-GATCAGCCCGAGGTTATCTA. The sizes of the PCR products were confirmed by gel electrophoresis and the specificity of the reaction was confirmed by melting curve analysis of the PCR products. For statistical analysis, the unpaired *t*-test was used.

### Antibodies and immunohistochemistry.

A polyclonal antibody against human collagen XXIX protein was raised by immunizing rabbits with a collagen XXIX specific peptide (SLGSTRKDDMEELAC, residues 2115–2128) (Eurogentec; http://www.eurogentec.com). The specificity of the antibodies purified by affinity chromatography was tested by comparing their reactivity against recombinant proteins by Western blotting and by blocking experiments.

For immunohistochemistry, freshly isolated skin specimens from five AD patients and five healthy individuals were embedded in Tissue-Tek. Cryosections of 5 μm thickness were prepared and fixed with acetone. Sections were incubated with anti-collagen XXIX antibodies followed by dextran-coupled anti-rabbit antibody, an alkaline phosphatase labelled amplification polymer (DAKO EnVision System; http://www.dako.com) and detection with fuchsin (DAKO). Nuclei were counterstained with Mayer's hematoxylin solution (Sigma-Aldrich; http://www.sigmaaldrich.com). Omission of primary antibody and preincubation with equimolar amount of peptide used for generation of the antibody in rabbits were used as negative controls for parallel sections. The results were consistent among the AD patients on one hand, and among the control biopsies on the other.

## Supporting Information

Figure S1 Hapmap LD Data for the *COL29A1* Region on Human Chromosome 3 LD plot for the CEPH population (Utah residents with ancestry from northern and western Europe) is shown [19]. The intensity of the color of the red boxes represents the pairwise LD values (r^2^) between two markers. Positions of the markers genotyped by the HapMap Project are indicated by vertical dashes on the horizontal bar. The scale indicates the physical position on Chromosome 3 in kb. Gene loci were taken from the human genome NCBI build 36.2 and correspond to the largest mRNA identified (FLJ35880 and LOC131873) or were predicted (LOC646300).
(1.3 MB TIF)

Figure S2 Gene Expression Analysis Gene expression analysis of *COL29A1* in the epidermis of AD patients (A) and of non-affected individuals (B). Cryosections (5 μm thick) of skin biopsies of five AD patients (AD1 to AD5) and of five normal human control individuals (NC1 to NC5) were hybridized with a digoxigenin-labeled antisense probe specific for *COL29A1* mRNA (on the left) and with a digoxigenin-labeled sense probe as a negative control (on the right). Staining of the detected transcripts was performed with the BCIP/NBT staining solution (blue). No staining was observed in the upper spinous and granular layer (indicated by arrows) of AD patients. The mRNA expression pattern was in good agreement with the results of the immunohistochemical staining of the protein. Sections were photographed at 200×magnification (AD5, 100×).
(15 MB TIF)

Figure S3 Immunohistochemical Analysis Immunohistochemical analysis of collagen XXIX expression in the epidermis of AD patients (A) and of non-affected individuals (B). Cryosections (5 μm thick) of skin biopsies of five AD patients (AD1–AD4, AD6) and of five normal human control individuals (NC1–NC3, NC6, NC7) were incubated with a rabbit polyclonal antibody against ColXXIX epitope and stained with fuchsin (red). Counterstaining was performed with hematoxylin (blue). Negative controls (on the right) were lacking the primary antibody. In normal, human skin, collagen XXIX is expressed in the epidermis with a slight increase towards the more differentiated layers. AD patients lack the protein in the upper spinous and granular layer as indicated by arrows. Sections were photographed at 200× magnification (AD5, 100×).
(18 MB TIF)

Table S1 SNPs Used in the Association Scan in the 5.4-Mb Region Between Markers *M3CS075* and *M3CS233*
The average typing rate was ≥ 97.9%
(564 KB DOC)

Table S2 Nonsynonymous Coding SNPs in *COL29A1*
vWA, von Willebrand factor domain; Col, collagen domain
(113 KB DOC)

Table S3 Results of the ASPEX sib_TDT for SNPs in the Region of *COL29A1* for Allergic Sensitization in the Discovery Cohort (112 KB DOC)

Table S4 Association Analysis of Single Markers for Allergic Sensitization in the Replication Dataset (58 KB DOC)

Table S5 Haplotype Analysis for Allergic Sensitization in the Replication Dataset (49 KB DOC)

### Accession Numbers

The GenBank (http://www.ncbi.nlm.nih.gov/Genbank/index.html) accession numbers for genes and proteins discussed in this paper are: AK093199 *(FLJ35880),* NM 153264 *(FLJ35880)*, and NP 476507 (human collagen VI alpha 3 chain),

The Protein Databank (http://www.pdb.com) accession number for the human vWF A3 domain is 1atz.

## Abbreviations

AD: atopic dermatitis
cM: centiMorgan
*COL29A1*: collagen XXIX gene
ECM: extracellular matrix
LD: linkage disequilibrium
RT-PCR: reverse-transcriptase PCR
SNP: single nucleotide polymorphism
vWA: von Willebrand factor–type A domain
