# Supplementary material for: Variants in a Novel Epidermal Collagen Gene (COL29A1) Are Associated with Atopic Dermatitis
Source: PLoS Biol. 2007 Sep 11;5(9):e242. doi: 10.1371/journal.pbio.0050242 (PMC1971127; doi:10.1371/journal.pbio.0050242)
Supplement: Supplementary file 1 [file pbio.0050242.st001.doc]

##### Supporting Material

**Table S1**

SNPs used in the association scan in the 5,4 Mb region between markers M3CS075 and M3CS233. The average typing rate was ≥ 97,9%.

|  | SNP | Chromosome 3 | MAF | Major/ |  | Atopic dermatitis | | |  | Allergic sensitization | | |
| --- | --- | --- | --- | --- | --- | --- | --- | --- | --- | --- | --- | --- |
|  |  | NCBI Build 36.1 |  | Minor  allele |  | All  P value | Paternal  P value | Maternal  P value |  | Paternal  P value | Maternal  P value | All  P value |
| 1 | rs1532602 | 126,107,810 | 42.7 | C/T |  | 0.97 | 0.21 | 0.33 |  | 0.95 | 0.34 | 0.3 |
| 2 | rs2270780 | 126,114,817 | 22.7 | A/G |  | 0.42 | 0.23 | 0.82 |  | 0.57 | 0.22 | 1 |
| 3 | rs2332924 | 126,127,555 | 24 | A/G |  | 0.35 | 0.039 | 0.68 |  | 0.71 | 0.11 | 0.31 |
| 4 | pos205149 | 126,277,468 | 9.4 | C/G |  | 0.22 | 0.65 | 0.29 |  | 0.27 | 0.64 | 0.36 |
| 5 | rs2333034 | 126,277,567 | 25.3 | G/T |  | 0.068 | 0.12 | 0.15 |  | 0.099 | 0.068 | 0.32 |
| 6 | rs2076729 | 126,278,274 | 35.5 | T/C |  | 0.13 | 0.71 | 0.025 |  | 0.31 | 0.92 | 0.089 |
| 7 | rs2981482 | 126,285,578 | 47 | A/G |  | 0.02 | 0.043 | 0.065 |  | 0.051 | 0.073 | 0.17 |
| 8 | rs651630 | 126,294,004 | 43.7 | A/G |  | 0.67 | 0.3 | 0.9 |  | 0.4 | 0.57 | 0.38 |
| 9 | rs2076727 | 126,299,784 | 47.2 | A/G |  | 0.12 | 0.11 | 0.36 |  | 0.25 | 0.12 | 0.74 |
| 10 | rs13325275 | 126,303,160 | 8.3 | G/A |  | 0.054 | 0.2 | 0.079 |  | 0.17 | 0.62 | 0.11 |
| 11 | rs12487908 | 126,313,738 | 15.7 | C/T |  | 0.58 | 0.43 | 0.8 |  | 0.81 | 0.32 | 0.73 |
| 12 | rs591803 | 126,318,469 | 29.4 | G/A |  | 0.92 | 0.55 | 0.79 |  | 1 | 0.24 | 0.33 |
| 13 | rs1554241 | 126,323,002 | 37.2 | T/A |  | 0.073 | 0.18 | 0.091 |  | 0.25 | 0.41 | 0.29 |
| 14 | rs12492332 | 126,324,170 | 16.8 | C/T |  | 0.42 | 0.38 | 0.72 |  | 0.64 | 0.31 | 0.85 |
| 15 | rs1971408 | 126,328,091 | 34.5 | T/A |  | 0.43 | 0.2 | 0.83 |  | 0.67 | 0.26 | 0.94 |
| 16 | rs516355 | 126,330,592 | 50 | A/C |  | 0.57 | 0.85 | 0.45 |  | 1 | 0.86 | 0.83 |
| 17 | rs2228677 | 126,331,445 | 48.4 | A/G |  | 0.89 | 0.69 | 0.49 |  | 0.48 | 1 | 0.31 |
| 18 | rs702045 | 126,332,460 | 10.3 | A/G |  | 0.67 | 0.32 | 0.73 |  | 0.65 | 0.18 | 0.53 |
| 19 | rs2981503 | 126,337,326 | 35.8 | T/A |  | 0.79 | 0.41 | 0.86 |  | 1 | 0.6 | 0.69 |
| 20 | rs504255 | 126,338,381 | 20.5 | C/T |  | 0.45 | 0.26 | 0.86 |  | 0.86 | 0.21 | 0.44 |
| 21 | rs531740 | 126,339,080 | 19.8 | A/G |  | 0.87 | 0.7 | 1 |  | 1 | 0.44 | 0.55 |
| 22 | rs6765325 | 126,345,375 | 18.2 | C/T |  | 0.55 | 0.44 | 0.79 |  | 0.68 | 0.35 | 0.84 |
| 23 | rs631691 | 126,357,868 | 50 | T/C |  | 0.48 | 0.57 | 0.098 |  | 0.37 | 0.6 | 0.063 |
| 24 | rs1398759 | 126,371,595 | 38.4 | G/C |  | 0.29 | 0.51 | 0.21 |  | 0.3 | 0.77 | 0.093 |
| 25 | rs2993631 | 126,379,358 | 5.3 | G/A |  | 0.5 | 0.32 | 0.9 |  | 0.3 | 0.23 | 0.46 |
| 26 | a304958 | 126,389,178 | 19.4 | A/T |  | 0.36 | 0.82 | 0.12 |  | 0.084 | 0.55 | 0.042 |
| 27 | c171945 | 126,391,761 | 1.3 | C/T |  | 0.18 | 0.26 | 0.62 |  | 0.25 | 0.31 | 0.61 |
| 28 | rs1980079 | 126,395,237 | 38.9 | A/G |  | 0.91 | 0.95 | 0.73 |  | 0.14 | 0.69 | 0.052 |
| 29 | rs2137599 | 126,397,335 | 38.9 | A/G |  | 0.87 | 1 | 0.76 |  | 0.13 | 0.64 | 0.05 |
| 30 | a1486438 | 126,407,361 | 11 | A/C |  | 0.58 | 0.49 | 0.81 |  | 0.23 | 0.46 | 0.3 |
| 31 | rs6778311 | 126,410,481 | 40.4 | T/C |  | 0.7 | 0.56 | 0.83 |  | 0.38 | 0.53 | 0.32 |
| 32 | c194270 | 126,413,098 | 44 | G/A |  | 0.97 | 0.48 | 0.56 |  | 0.36 | 0.22 | 0.62 |
| 33 | rs655465 | 126,414,557 | 43.7 | A/C |  | 0.55 | 0.77 | 0.2 |  | 1 | 0.47 | 0.51 |
| 34 | rs1053738 | 126,434,511 | 24.1 | T/C |  | 0.18 | 0.21 | 0.41 |  | 0.055 | 0.073 | 0.24 |
| 35 | rs1850506 | 126,448,618 | 24.1 | C/T |  | 0.51 | 0.49 | 0.62 |  | 0.25 | 0.29 | 0.52 |
| 36 | pos400840 | 126,473,159 | 10.5 | T/C |  | 0.2 | 0.62 | 0.076 |  | 0.51 | 0.6 | 0.4 |
| 37 | rs1999921 | 126,473,528 | 24.4 | A/G |  | 0.34 | 0.45 | 0.52 |  | 0.13 | 0.13 | 0.39 |
| 38 | rs7615139 | 126,476,877 | 24.2 | T/C |  | 0.41 | 0.46 | 0.61 |  | 0.16 | 0.14 | 0.46 |
| 39 | rs1975760 | 126,513,071 | 24.6 | C/T |  | 0.39 | 0.45 | 0.48 |  | 0.24 | 0.22 | 0.47 |
| 40 | a1486427 | 126,522,281 | 31.1 | C/A |  | 0.15 | 0.33 | 0.2 |  | 0.39 | 0.76 | 0.27 |
| 41 | rs4679167 | 126,525,957 | 24.2 | C/A |  | 0.41 | 0.46 | 0.51 |  | 0.16 | 0.16 | 0.41 |
| 42 | rs12485473 | 126,532,626 | 40.4 | C/T |  | 0.14 | 0.23 | 0.22 |  | 0.3 | 0.15 | 0.82 |
| 43 | rs2076725 | 126,604,771 | 36.4 | T/C |  | 0.91 | 0.31 | 0.61 |  | 0.46 | 0.15 | 0.93 |
| 44 | rs1839063 | 126,726,432 | 18.7 | G/T |  | 0.3 | 0.78 | 0.16 |  | 0.48 | 1 | 0.21 |
| 45 | rs2294053 | 126,732,030 | 9.3 | G/A |  | 0.32 | 0.069 | 0.77 |  | 0.35 | 0.017 | 0.52 |
| 46 | rs2922172 | 126,770,206 | 23.3 | A/G |  | 0.18 | 0.3 | 0.21 |  | 0.25 | 0.29 | 0.42 |
| 47 | pos698044 | 126,770,363 | 9 | G/A |  | 0.75 | 0.15 | 0.41 |  | 0.79 | 0.049 | 0.31 |
| 48 | pos708682 | 126,781,001 | 14.1 | C/T |  | 0.3 | 0.32 | 0.59 |  | 0.78 | 0.45 | 0.84 |
| 49 | rs3829007 | 126,781,202 | 9.2 | T/C |  | 0.22 | 0.044 | 0.92 |  | 0.21 | 0.024 | 0.91 |
| 50 | rs2979306 | 126,794,820 | 13.7 | C/A |  | 1 | 0.85 | 0.76 |  | 0.82 | 0.81 | 0.83 |
| 51 | rs2976713 | 126,882,374 | 13.1 | A/C |  | 0.8 | 0.61 | 0.83 |  | 1 | 0.64 | 0.71 |
| 52 | rs12633485 | 127,088,267 | 17.9 | C/T |  | 0.36 | 0.52 | 0.3 |  | 0.12 | 0.16 | 0.2 |
| 53 | rs10934738 | 127,162,739 | 40.2 | G/C |  | 0.73 | 0.85 | 0.62 |  | 0.77 | 0.53 | 0.32 |
| 54 | rs11925047 | 127,181,634 | 27 | G/A |  | 0.54 | 0.5 | 0.65 |  | 0.46 | 0.68 | 0.34 |
| 55 | rs11543284 | 127,208,145 | 10 | G/C |  | 0.21 | 0.44 | 0.19 |  | 0.38 | 0.78 | 0.32 |
| 56 | rs1044215 | 127,208,249 | 20.2 | C/G |  | 0.054 | 0.39 | 0.034 |  | 0.01 | 0.2 | 0.01 |
| 57 | rs2279820 | 127,216,962 | 26.7 | G/A |  | 1 | 0.94 | 0.93 |  | 0.72 | 1 | 0.49 |
| 58 | rs2279821 | 127,216,998 | 20 | G/A |  | 0.074 | 0.34 | 0.059 |  | 0.013 | 0.15 | 0.011 |
| 59 | rs868149 | 127,233,547 | 25.8 | C/A |  | 0.79 | 0.55 | 0.89 |  | 0.49 | 0.54 | 0.66 |
| 60 | rs1503073 | 127,241,922 | 27 | G/C |  | 0.69 | 0.72 | 0.69 |  | 0.65 | 0.94 | 0.49 |
| 61 | rs4077563 | 127,252,447 | 32.2 | C/T |  | 0.77 | 0.8 | 0.46 |  | 0.73 | 0.5 | 0.93 |
| 62 | rs4449265 | 127,262,342 | 31.8 | G/A |  | 0.45 | 0.63 | 0.45 |  | 0.69 | 0.85 | 0.45 |
| 63 | rs4493383 | 127,266,380 | 33.2 | T/C |  | 0.84 | 0.71 | 1 |  | 0.92 | 1 | 0.82 |
| 64 | c32184851 | 127,268,005 | 32.6 | A/G |  | 0.84 | 0.67 | 1 |  | 1 | 0.86 | 0.81 |
| 65 | c32197449 | 127,280,603 | 28.6 | G/A |  | 0.77 | 0.59 | 0.94 |  | 0.46 | 0.77 | 0.3 |
| 66 | rs4646760 | 127,305,561 | 39.7 | A/G |  | 0.56 | 0.77 | 0.22 |  | 0.49 | 0.87 | 0.19 |
| 67 | rs2276729 | 127,308,904 | 19.2 | G/A |  | 0.21 | 0.5 | 0.17 |  | 0.047 | 0.22 | 0.066 |
| 68 | rs2305230 | 127,339,385 | 15.5 | C/A |  | 0.094 | 0.12 | 0.3 |  | 0.58 | 0.41 | 0.82 |
| 69 | rs1868121 | 127,388,343 | 36.2 | C/T |  | 0.11 | 0.2 | 0.15 |  | 0.32 | 0.33 | 0.47 |
| 70 | rs747037 | 127,475,849 | 6.1 | C/A |  | 0.58 | 1 | 0.28 |  | 0.74 | 0.53 | 0.19 |
| 71 | pos1473510 | 127,545,819 | 12.7 | C/G |  | 0.68 | 1 | 0.46 |  | 0.95 | 0.9 | 0.74 |
| 72 | rs777485 | 127,689,359 | 26.9 | G/C |  | 0.68 | 0.29 | 0.73 |  | 0.87 | 0.73 | 0.4 |
| 73 | rs777484 | 127,689,405 | 47.7 | A/C |  | 0.09 | 0.14 | 0.16 |  | 0.3 | 0.42 | 0.34 |
| 74 | rs1056522 | 127,744,035 | 29.9 | G/A |  | 0.53 | 0.64 | 0.47 |  | 0.94 | 0.64 | 0.52 |
| 75 | rs3732534 | 127,823,243 | 37.5 | A/G |  | 0.1 | 0.032 | 0.55 |  | 0.11 | 0.0033 | 0.95 |
| 76 | rs4679128 | 127,863,494 | 22.5 | G/A |  | 0.096 | 0.024 | 0.71 |  | 0.59 | 0.071 | 0.42 |
| 77 | rs2053820 | 127,977,996 | 34 | T/C |  | 0.27 | 0.54 | 0.21 |  | 0.42 | 0.45 | 0.53 |
| 78 | rs1014081 | 127,982,798 | 33.9 | G/A |  | 0.16 | 0.45 | 0.12 |  | 0.53 | 0.64 | 0.55 |
| 79 | rs17693923 | 127,999,896 | 23.8 | T/G |  | 0.14 | 0.089 | 0.55 |  | 0.78 | 0.49 | 0.79 |
| 80 | rs1542234 | 128,034,762 | 9.7 | G/A |  | 1 | 0.63 | 0.61 |  | 0.31 | 0.56 | 0.25 |
| 81 | rs11714878 | 128,056,685 | 13.1 | T/C |  | 0.31 | 0.13 | 0.93 |  | 0.079 | 0.018 | 0.51 |
| 82 | rs7633939 | 128,091,288 | 27.6 | A/G |  | 0.26 | 0.41 | 0.33 |  | 0.47 | 0.55 | 0.63 |
| 83 | rs1008175 | 128,102,704 | 18.1 | G/A |  | 0.82 | 0.42 | 0.68 |  | 0.31 | 0.93 | 0.079 |
| 84 | rs9683346 | 128,116,700 | 32.2 | A/G |  | 0.21 | 0.33 | 0.29 |  | 0.47 | 0.48 | 0.7 |
| 85 | rs734506 | 128,127,236 | 32 | A/G |  | 0.19 | 0.27 | 0.32 |  | 0.51 | 0.47 | 0.75 |
| 86 | rs4679309 | 128,148,697 | 31.2 | A/G |  | 0.17 | 0.35 | 0.18 |  | 0.42 | 0.38 | 0.62 |
| 87 | rs6777746 | 128,160,859 | 13.2 | T/C |  | 0.45 | 0.23 | 0.94 |  | 0.097 | 0.033 | 0.45 |
| 88 | rs9864643 | 128,186,164 | 8.6 | A/G |  | 0.63 | 0.36 | 0.91 |  | 0.56 | 0.77 | 0.49 |
| 89 | rs1347003 | 128,214,737 | 27.6 | A/G |  | 0.25 | 0.3 | 0.44 |  | 0.53 | 0.64 | 0.6 |
| 90 | rs2241675 | 128,231,707 | 8.4 | T/C |  | 0.94 | 0.75 | 1 |  | 0.16 | 0.22 | 0.34 |
| 91 | rs900422 | 128,350,249 | 31.1 | T/C |  | 0.28 | 0.12 | 0.82 |  | 0.46 | 0.31 | 0.75 |
| 92 | rs977683 | 128,396,814 | 26.2 | C/G |  | 0.63 | 0.32 | 0.95 |  | 0.68 | 0.84 | 0.4 |
| 93 | rs6776267 | 128,501,229 | 40.1 | G/A |  | 0.097 | 0.063 | 0.41 |  | 0.28 | 0.14 | 0.61 |
| 94 | rs6776659 | 128,501,436 | 21.3 | G/A |  | 0.1 | 0.026 | 0.63 |  | 0.39 | 0.1 | 1 |
| 95 | rs6789659 | 128,501,526 | 18 | A/T |  | 0.58 | 0.8 | 0.41 |  | 0.14 | 0.55 | 0.088 |
| 96 | rs1001942 | 128,614,211 | 34.3 | A/C |  | 0.88 | 0.88 | 0.91 |  | 0.63 | 0.73 | 0.58 |
| 97 | rs1072711 | 128,670,101 | 21.3 | G/A |  | 0.95 | 1 | 0.83 |  | 0.45 | 0.28 | 0.72 |
| 98 | rs2720251 | 128,703,677 | 17 | T/C |  | 0.68 | 0.26 | 0.7 |  | 0.74 | 0.36 | 0.79 |
| 99 | rs2720244 | 128,709,235 | 17 | T/C |  | 0.71 | 0.18 | 0.64 |  | 0.74 | 0.32 | 0.73 |
| 100 | rs2720240 | 128,730,064 | 16.7 | A/G |  | 0.64 | 0.2 | 0.63 |  | 0.73 | 0.33 | 0.73 |
| 101 | rs768853 | 128,747,382 | 47.1 | A/G |  | 0.14 | 0.11 | 0.42 |  | 0.081 | 0.15 | 0.12 |
| 102 | rs768854 | 128,747,599 | 24.5 | C/T |  | 0.76 | 0.71 | 0.38 |  | 0.6 | 0.87 | 0.27 |
| 103 | rs893293 | 128,806,520 | 25.5 | T/C |  | 0.27 | 0.94 | 0.1 |  | 0.75 | 0.5 | 0.2 |
| 104 | rs782454 | 128,875,420 | 24.6 | A/G |  | 0.43 | 0.89 | 0.16 |  | 0.85 | 0.42 | 0.34 |
| 105 | rs1658037 | 128,878,910 | 24.8 | C/T |  | 0.24 | 0.81 | 0.075 |  | 0.71 | 0.43 | 0.21 |
| 106 | rs782456 | 128,880,866 | 25 | G/A |  | 0.13 | 0.57 | 0.048 |  | 0.38 | 0.85 | 0.16 |
| 107 | rs782441 | 128,895,263 | 20.8 | C/T |  | 1 | 0.59 | 0.59 |  | 0.6 | 0.85 | 0.27 |
| 108 | rs782440 | 128,898,832 | 45.9 | C/T |  | 0.58 | 0.21 | 0.74 |  | 1 | 0.49 | 0.55 |
| 109 | rs782438 | 128,899,738 | 36 | C/T |  | 0.34 | 0.26 | 0.69 |  | 0.96 | 0.62 | 0.77 |
| 110 | rs782451 | 128,910,158 | 46.3 | C/G |  | 0.62 | 0.26 | 0.73 |  | 1 | 0.47 | 0.53 |
| 111 | rs1437553 | 128,910,282 | 41.8 | C/A |  | 0.88 | 0.48 | 0.71 |  | 1 | 0.62 | 0.64 |
| 112 | rs1010174 | 128,912,257 | 32.3 | C/G |  | 0.33 | 0.57 | 0.32 |  | 0.72 | 0.79 | 0.34 |
| 113 | rs3821581 | 128,912,615 | 42.3 | G/A |  | 0.84 | 0.78 | 0.56 |  | 0.54 | 0.73 | 0.48 |
| 114 | rs3773131 | 128,912,668 | 9.3 | C/G |  | 0.61 | 0.27 | 0.83 |  | 1 | 0.66 | 0.68 |
| 115 | rs3773135 | 128,914,091 | 41.6 | C/T |  | 1 | 0.73 | 0.72 |  | 0.77 | 1 | 0.57 |
| 116 | rs3773136 | 128,914,119 | 10 | T/A |  | 0.79 | 0.22 | 0.35 |  | 1 | 0.33 | 0.36 |
| 117 | rs3773137 | 128,914,333 | 32.4 | G/A |  | 0.47 | 0.73 | 0.42 |  | 0.92 | 0.51 | 0.76 |
| 118 | rs597956 | 128,915,249 | 10.4 | G/A |  | 1 | 0.4 | 0.39 |  | 1 | 0.54 | 0.53 |
| 119 | rs534018 | 128,916,232 | 47.7 | C/T |  | 0.81 | 0.64 | 0.29 |  | 0.64 | 1 | 0.44 |
| 120 | rs477554 | 128,917,546 | 47.3 | C/T |  | 1 | 0.6 | 0.55 |  | 0.85 | 0.94 | 0.68 |
| 121 | rs479504 | 128,917,790 | 19.7 | G/T |  | 1 | 1 | 0.92 |  | 0.71 | 0.73 | 0.62 |
| 122 | rs3773146 | 128,917,881 | 43.2 | C/T |  | 0.76 | 0.32 | 0.6 |  | 1 | 0.55 | 0.48 |
| 123 | rs3773154 | 128,921,215 | 17.9 | C/T |  | 0.63 | 0.89 | 0.53 |  | 0.42 | 0.42 | 0.63 |
| 124 | rs3773155 | 128,921,218 | 11.2 | A/G |  | 1 | 0.46 | 0.6 |  | 0.92 | 0.38 | 0.64 |
| 125 | rs9811688 | 128,921,577 | 32 | T/C |  | 1 | 0.79 | 0.72 |  | 0.57 | 0.47 | 0.88 |
| 126 | rs526862 | 128,922,071 | 45.7 | A/G |  | 1 | 0.31 | 0.27 |  | 0.61 | 0.88 | 0.32 |
| 127 | rs555183 | 128,922,832 | 45.8 | A/G |  | 0.96 | 0.41 | 0.31 |  | 0.83 | 0.72 | 0.49 |
| 128 | rs1466571 | 128,936,220 | 32.1 | G/A |  | 0.66 | 0.74 | 0.61 |  | 0.96 | 0.71 | 0.86 |
| 129 | rs1046821 | 128,945,878 | 41.4 | C/T |  | 0.81 | 0.89 | 0.62 |  | 0.57 | 0.31 | 0.94 |
| 130 | rs11915484 | 128,956,517 | 17.1 | G/A |  | 0.35 | 0.76 | 0.24 |  | 0.36 | 0.53 | 0.45 |
| 131 | rs664910 | 128,956,720 | 28.3 | A/G |  | 0.38 | 1 | 0.15 |  | 0.75 | 0.77 | 0.36 |
| 132 | pos480745 | 128,964,601 | 12.7 | G/T |  | 0.38 | 1 | 0.12 |  | 0.29 | 0.69 | 0.16 |
| 133 | rs549662 | 128,964,726 | 17.1 | A/G |  | 0.49 | 0.37 | 0.71 |  | 0.83 | 0.74 | 0.91 |
| 134 | rs541042 | 128,967,121 | 13.3 | T/C |  | 0.83 | 0.45 | 0.22 |  | 0.64 | 0.74 | 0.2 |
| 135 | rs567384 | 128,976,554 | 11.8 | T/C |  | 1 | 0.75 | 0.85 |  | 0.73 | 0.46 | 0.19 |
| 136 | rs587779 | 128,978,605 | 14.2 | T/C |  | 1 | 0.85 | 0.91 |  | 0.86 | 0.49 | 0.71 |
| 137 | rs541855 | 128,978,766 | 17.6 | C/T |  | 1 | 0.33 | 0.27 |  | 1 | 0.9 | 0.82 |
| 138 | rs586919 | 128,978,778 | 17.5 | G/C |  | 1 | 0.33 | 0.27 |  | 0.92 | 0.9 | 0.71 |
| 139 | rs9289321 | 129,011,459 | 15.7 | G/A |  | 0.73 | 0.66 | 0.82 |  | 0.62 | 0.81 | 0.35 |
| 140 | rs1543272 | 129,070,913 | 10.7 | G/A |  | 0.95 | 0.43 | 0.2 |  | 0.66 | 0.92 | 0.28 |
| 141 | rs1014470 | 129,113,596 | 6.9 | G/C |  | 0.41 | 1 | 0.087 |  | 0.55 | 0.7 | 0.11 |
| 142 | rs2217628 | 129,134,319 | 14.4 | C/T |  | 0.53 | 0.73 | 0.42 |  | 0.8 | 1 | 0.64 |
| 143 | rs1469656 | 129,161,494 | 45.8 | A/G |  | 1 | 0.9 | 0.83 |  | 0.67 | 0.73 | 0.73 |
| 144 | rs4857926 | 129,192,982 | 34.4 | C/T |  | 0.91 | 0.89 | 0.95 |  | 0.26 | 0.28 | 0.47 |
| 145 | rs1042907 | 129,271,862 | 26.6 | C/G |  | 0.69 | 0.58 | 0.22 |  | 0.66 | 0.46 | 0.22 |
| 146 | rs7632756 | 129,300,198 | 26.3 | T/C |  | 0.19 | 0.62 | 0.11 |  | 0.26 | 0.78 | 0.084 |
| 147 | rs2955114 | 129,360,446 | 16.6 | A/C |  | 0.29 | 0.87 | 0.084 |  | 0.16 | 0.85 | 0.032 |
| 148 | rs6777054 | 129,428,039 | 25.8 | A/G |  | 0.17 | 0.47 | 0.12 |  | 0.3 | 0.73 | 0.17 |
| 149 | rs2955103 | 129,497,926 | 37.3 | T/C |  | 0.13 | 0.9 | 0.018 |  | 0.81 | 0.46 | 0.25 |
| 150 | rs1735538 | 129,574,792 | 23.7 | A/G |  | 0.21 | 0.57 | 0.15 |  | 0.41 | 0.78 | 0.23 |
| 151 | rs2981015 | 129,672,076 | 22.3 | A/G |  | 0.92 | 0.28 | 0.16 |  | 0.68 | 0.83 | 0.39 |
| 152 | rs3803 | 129,682,070 | 16.5 | G/A |  | 0.23 | 0.53 | 0.15 |  | 0.24 | 0.62 | 0.21 |
| 153 | rs2713603 | 129,683,224 | 39.8 | G/A |  | 0.48 | 0.85 | 0.27 |  | 0.73 | 0.71 | 0.71 |
| 154 | rs2860228 | 129,692,357 | 44.9 | C/T |  | 0.42 | 0.081 | 0.75 |  | 0.047 | 0.038 | 0.18 |
| 155 | rs11712335 | 129,693,240 | 27.8 | T/C |  | 0.48 | 0.53 | 0.53 |  | 0.056 | 0.26 | 0.048 |
| 156 | rs4857914 | 129,833,374 | 35.1 | C/T |  | 0.63 | 1 | 0.36 |  | 0.32 | 0.4 | 0.38 |
| 157 | a488884 | 129,947,416 | 18.5 | G/C |  | 0.12 | 0.17 | 0.27 |  | 0.14 | 0.35 | 0.17 |
| 158 | rs7642199 | 129,998,911 | 18.5 | A/G |  | 0.5 | 0.79 | 0.4 |  | 0.67 | 0.61 | 0.65 |
| 159 | rs789230 | 130,085,248 | 23.5 | G /A |  | 0.18 | 0.081 | 0.68 |  | 0.5 | 0.096 | 0.7 |
| 160 | rs883602 | 130,136,642 | 25.3 | A/G |  | 0.032 | 0.042 | 0.16 |  | 0.23 | 0.05 | 0.8 |
| 161 | rs12695512 | 130,204,772 | 43.6 | G/T |  | 0.74 | 0.27 | 0.7 |  | 0.53 | 0.19 | 0.88 |
| 162 | rs11709852 | 130,389,647 | 12.2 | G/A |  | 0.52 | 0.23 | 1 |  | 0.59 | 0.51 | 0.62 |
| 163 | rs140695 | 130,634,834 | 28 | C/T |  | 0.82 | 1 | 0.69 |  | 0.73 | 0.8 | 0.73 |
| 164 | a3140797 | 130,706,883 | 9.5 | C/T |  | 0.63 | 0.54 | 0.14 |  | 0.71 | 0.63 | 0.22 |
| 165 | rs2625967 | 130,749,949 | 9.9 | G/A |  | 0.74 | 1 | 0.43 |  | 1 | 0.57 | 0.7 |
| 166 | rs2255703 | 130,775,946 | 36.8 | T/C |  | 0.67 | 0.78 | 0.69 |  | 0.64 | 0.82 | 0.29 |
| 167 | rs10804590 | 130,816,761 | 17 | C/T |  | 0.8 | 0.85 | 0.8 |  | 1 | 1 | 0.92 |
| 168 | rs2717250 | 131,057,162 | 14.8 | A/G |  | 0.75 | 0.58 | 0.86 |  | 0.59 | 0.33 | 1 |
| 169 | rs9289347 | 131,087,680 | 14.2 | A/G |  | 0.8 | 0.53 | 0.93 |  | 0.76 | 0.31 | 0.7 |
| 170 | rs938194 | 131,156,190 | 22.3 | C/G |  | 0.59 | 0.73 | 0.62 |  | 0.64 | 0.62 | 0.22 |
| 171 | rs7630899 | 131,167,973 | 42.5 | C/T |  | 0.76 | 0.94 | 0.52 |  | 0.38 | 0.87 | 0.11 |
| 172 | rs6791923 | 131,189,082 | 39.8 | A/G |  | 0.63 | 0.69 | 0.65 |  | 0.64 | 0.51 | 0.83 |
| 173 | rs9836814 | 131,417,587 | 28.8 | A/G |  | 0.15 | 0.57 | 0.1 |  | 0.091 | 0.13 | 0.22 |
| 174 | rs6802606 | 131,422,477 | 13 | G/A |  | 0.69 | 0.84 | 0.65 |  | 0.48 | 1 | 0.31 |
| 175 | rs2896565 | 131,432,956 | 7.8 | G/A |  | 0.36 | 1 | 0.15 |  | 0.57 | 0.28 | 0.13 |
| 176 | rs9871734 | 131,442,289 | 21.1 | A/G |  | 0.51 | 0.62 | 0.48 |  | 0.46 | 0.097 | 0.76 |
| 177 | a36458361 | 131,445,897 | 1.1 | G/A |  | 0.37 | 1 | 0.5 |  | 0.77 | 1 | 1 |
| 178 | rs6777871 | 131,452,507 | 10.9 | G/A |  | 0.17 | 0.64 | 0.065 |  | 0.69 | 0.31 | 0.19 |
| 179 | rs6804874 | 131,458,738 | 20.6 | C/A |  | 0.32 | 0.48 | 0.36 |  | 0.14 | 0.046 | 0.7 |
| 180 | rs1453255 | 131,460,348 | 32 | G/A |  | 0.084 | 0.11 | 0.18 |  | 0.19 | 0.11 | 0.43 |
| 181 | rs9832769 | 131,468,386 | 15.4 | A/G |  | 0.054 | 0.32 | 0.026 |  | 0.44 | 0.65 | 0.11 |
| 182 | a25930206 | 131,468,667 | 4.6 | G/C |  | 0.46 | 0.27 | 0.67 |  | 0.57 | 0.42 | 0.62 |
| 183 | a36487080 | 131,474,616 | 4.5 | A/G |  | 0.28 | 0.19 | 0.5 |  | 0.33 | 0.27 | 0.44 |
| 184 | a36487277 | 131,474,813 | 9.1 | C/T |  | 0.12 | 0.42 | 0.11 |  | 0.1 | 0.12 | 0.55 |
| 185 | rs7648240 | 131,479,302 | 10.9 | G/T |  | 1 | 0.89 | 0.92 |  | 0.93 | 0.89 | 0.89 |
| 186 | rs16825656 | 131,487,758 | 27.3 | A/G |  | 0.16 | 0.27 | 0.2 |  | 0.13 | 0.082 | 0.39 |
| 187 | rs6807290 | 131,499,485 | 26.7 | G/A |  | 0.16 | 0.28 | 0.25 |  | 0.11 | 0.081 | 0.35 |
| 188 | rs1349747 | 131,543,410 | 18 | A/G |  | 0.081 | 0.65 | 0.053 |  | 0.27 | 0.9 | 0.12 |
| 189 | rs5852593 | 131,580,335-131,580,336 | 24.3 | del/T |  | 0.0079 | 0.026 | 0.045 |  | 0.0082 | 0.018 | 0.095 |
| 190 | rs1497309 | 131,616,310 | 28.7 | C/T |  | 0.016 | 0.14 | 0.013 |  | 0.0055 | 0.042 | 0.011 |
| 191 | rs9883988 | 131,645,085 | 6.3 | G/A |  | 0.049 | 0.069 | 0.19 |  | 0.056 | 0.1 | 0.23 |
| 192 | rs819091 | 131,678,260 | 23.7 | G/A |  | 0.05 | 0.3 | 0.025 |  | 0.065 | 0.31 | 0.06 |
| 193 | rs1497319 | 131,708,045 | 29.4 | T/A |  | 0.065 | 0.3 | 0.037 |  | 0.07 | 0.35 | 0.045 |
| 194 | rs4682614 | 131,725,939 | 20.7 | G/A |  | 0.91 | 0.7 | 0.87 |  | 0.94 | 0.93 | 0.93 |
| 195 | rs9836961 | 131,769,576 | 16.4 | C/T |  | 0.19 | 0.15 | 0.5 |  | 0.28 | 0.29 | 0.42 |
| 196 | rs1508520 | 131,780,349 | 18.8 | T/C |  | 0.95 | 0.84 | 0.69 |  | 0.87 | 1 | 0.65 |
| 197 | rs6439249 | 131,783,340 | 11.8 | C/T |  | 0.67 | 0.34 | 0.78 |  | 0.88 | 0.48 | 0.71 |
| 198 | rs1027858 | 131,785,876 | 11.8 | A/C |  | 0.78 | 0.34 | 0.63 |  | 0.94 | 0.4 | 0.57 |
| 199 | rs9823913 | 131,829,269 | 20.6 | A/G |  | 0.52 | 0.88 | 0.23 |  | 0.65 | 0.85 | 0.42 |
| 200 | rs11710068 | 131,877,537 | 22.8 | C/T |  | 0.6 | 0.75 | 0.54 |  | 0.72 | 0.85 | 0.39 |
| 201 | rs12495186 | 131,881,158 | 22.6 | T/C |  | 0.46 | 0.79 | 0.32 |  | 0.8 | 0.63 | 0.32 |
| 202 | rs4682627 | 131,893,100 | 22.9 | T/C |  | 0.41 | 0.49 | 0.38 |  | 0.54 | 1 | 0.3 |
| 203 | rs900989 | 131,902,071 | 23.4 | A/G |  | 0.43 | 0.66 | 0.38 |  | 0.59 | 0.86 | 0.32 |
| 204 | rs10934955 | 131,933,044 | 23.5 | A/G |  | 0.5 | 0.72 | 0.43 |  | 0.6 | 0.86 | 0.31 |
| 205 | rs9824635 | 132,030,056 | 44.3 | G/T |  | 0.8 | 0.59 | 0.35 |  | 0.85 | 0.52 | 0.34 |
| 206 | rs712984 | 132,131,488 | 40.2 | G/A |  | 0.84 | 0.4 | 0.64 |  | 0.46 | 0.29 | 0.8 |
| 207 | rs218488 | 132,178,652 | 41.7 | C/T |  | 0.85 | 0.73 | 0.38 |  | 0.91 | 0.42 | 0.57 |
| 208 | rs218503 | 132,220,322 | 32.1 | G/A |  | 0.48 | 0.14 | 0.93 |  | 0.73 | 0.19 | 0.48 |
| 209 | rs17331416 | 132,279,027 | 20.4 | G/T |  | 1 | 0.27 | 0.24 |  | 0.95 | 0.13 | 0.16 |
| 210 | rs1400013 | 132,356,413 | 43.7 | G/T |  | 0.45 | 0.71 | 0.29 |  | 0.7 | 0.94 | 0.51 |
| 211 | rs6800866 | 132,424,091 | 21.5 | T/C |  | 0.43 | 0.87 | 0.23 |  | 0.61 | 0.85 | 0.3 |
| 212 | rs6439279 | 132,491,823 | 22.2 | C/T |  | 0.74 | 0.57 | 0.27 |  | 0.64 | 0.79 | 0.23 |
