# Supplementary material for: Variants in a Novel Epidermal Collagen Gene (COL29A1) Are Associated with Atopic Dermatitis
Source: PLoS Biol. 2007 Sep 11;5(9):e242. doi: 10.1371/journal.pbio.0050242 (PMC1971127; doi:10.1371/journal.pbio.0050242)
Supplement: Supplementary file 2 [file pbio.0050242.st002.doc]

**Table S2.** Association analysis of non-synonymous coding SNPs in *COL29A1* for atopic dermatitis in the discovery cohort.

|  |  |  |  |  |  |  |  | All | | | |  | Paternal | | | |  | Maternal | | | |
| --- | --- | --- | --- | --- | --- | --- | --- | --- | --- | --- | --- | --- | --- | --- | --- | --- | --- | --- | --- | --- | --- |
| SNP ID | Minor  allele % | Alleles | Amino acid exchange | Amino acid position | *COL29A1*  Location | Collagen XXIX Domain |  | T | NT | chi2 | P value^a^ |  | T | NT | chi2 | P value |  | T | NT | chi2 | P value |
| a36590333 | 1.9 | **T**/C | Met/Thr | 56 | exon 3 | VWFA1^d^ |  | 9 | 22 | 5.45 | 0.041 |  | 6 | 10 | 1 | 0.38 |  | 2 | 11 | 6.23 | 0.034 |
| rs1453241 | 24.0 | **G**/A | Glu/Lys | 455 | exon 5 | VWFA3 |  | 166 | 124 | 6.08 | 0.023 |  | 68 | 50 | 2.75 | 0.11 |  | 79 | 55 | 4.3 | 0.038 |
| a36602721 | 1.3 | **T**/G | Val/Gly | 669 | exon 6 | VWFA4 |  | 13 | 8 | 1.19 | 0.38 |  | 5 | 5 | 0 | 1 |  | 8 | 3 | 2.27 | 0.24 |
| rs16827168 | 1.9 | **A**/G | His/Arg | 805 | exon 6 |  |  | 16 | 14 | 0.13 | 0.84 |  | 7 | 6 | 0.08 | 1 |  | 9 | 8 | 0.06 | 1 |
| rs11917356 | 16.6 | **A**/G | Asp/Gly | 982 | exon 7 | VWFA5 |  | 121 | 99 | 2.2 | 0.19 |  | 50 | 40 | 1.11 | 0.32 |  | 59 | 47 | 1.36 | 0.25 |
| rs1353613 | <1.0 | **C**/G | Ile/Met | 1114 | exon 8 | VWFA6 |  | 4 | 4 | 0 | 1 |  |  |  |  |  |  |  |  |  |  |
| a36611838 | 7.8 | **G**/A | Val/Ile | 1276 | exon 9 |  |  | 57 | 54 | 0.08 | 0.87 |  | 31 | 23 | 1.19 | 0.36 |  | 23 | 28 | 0.49 | 0.53 |
| rs12488457 | 29.9 | **C**/A | Pro/Thr | 1280 | exon 9 |  |  | 201 | 155 | 5.94 | 0.025 |  | 82 | 62 | 2.78 | 0.11 |  | 85 | 59 | 4.69 | 0.042 |
| rs1497312 | 23.7 | **G**/C | Cys/Ser | 1477 | exon 17 | Col ^e^ |  | 162 | 137 | 2.09 | 0.2 |  | 56 | 52 | 0.15 | 0.7 |  | 74 | 53 | 3.47 | 0.077 |
| a36620315 | <1.0 | **C**/G | Ser/Cys | 1492 | exon 17 | Col |  | 6 | 7 | 0.08 | 1 |  |  |  |  |  |  |  |  |  |  |
| a36624476 | 13.5 | **C**/G | Thr/Ser | 1539 | exon 20 | Col |  | 109 | 83 | 3.52 | 0.086 |  | 50 | 44 | 0.38 | 0.59 |  | 50 | 30 | 5 | 0.027 |
| rs16827497 | 23.1 | **C**/T | Pro/Ser | 1589 | exon 23 | Col |  | 159 | 127 | 3.58 | 0.086 |  | 60 | 50 | 0.91 | 0.33 |  | 70 | 48 | 4.1 | 0.065 |
| a36645464 | 5.0 | **A**/C | Glu/Asp | 1750 | exon 33 |  |  | 55 | 38 | 3.11 | 0.078 |  | 30 | 20 | 2 | 0.16 |  | 20 | 13 | 1.48 | 0.29 |
| a36654694 | <1.0 | **C**/T | Arg/Cys | 2120 | exon 35 | VWFA 8 |  | 9 | 8 | 0.05 | 1 |  |  |  |  |  |  |  |  |  |  |
| rs9883988 | 6.3 | **G**/A | Arg/Gln | 2188 | exon 36 |  |  | 56 | 35 | 4.85 | 0.05 |  | 31 | 18 | 3.45 | 0.068 |  | 19 | 11 | 2.13 | 0.19 |
| rs819085 | 6.3 | **A**/G | Asp/Gly | 2205 | exon 37 |  |  | 56 | 35 | 4.85 | 0.049 |  | 31 | 18 | 3.45 | n.s. |  | 19 | 11 | 2.13 | n.s. |
| rs322117 | 18.2 | **A**/G | Asn/Ser | 2560 | exon 40 |  |  | 116 | 99 | 1.34 | 0.3 |  | 48 | 45 | 0.1 | 0.73 |  | 52 | 38 | 2.18 | 0.17 |
| a36685870 | 6.9 | **G**/A | Ala/Thr | 2589 | exon 40 |  |  | 54 | 46 | 0.64 | 0.52 |  | 25 | 22 | 0.19 | 0.71 |  | 26 | 21 | 0.53 | 0.49 |
| rs11355796 | 23.9 | _/T | Stop/Leu | 2591 | exon 40 |  |  | 133 | 109 | 2.38 | 0.16 |  | 56 | 52 | 0.15 | 0.73 |  | 54 | 34 | 4.55 | 0.031 |

^a^ *P* values of the ASPEX sib TDT for atopic dermatitis for all, paternal, and maternal transmissions.

^b^ T number of transmissions

^c^ NT number of non-transmissions

^d^ VWA von Willebrand factor domain

^e^ Col collagen domain
