# Supplementary material for: Variants in a Novel Epidermal Collagen Gene (COL29A1) Are Associated with Atopic Dermatitis
Source: PLoS Biol. 2007 Sep 11;5(9):e242. doi: 10.1371/journal.pbio.0050242 (PMC1971127; doi:10.1371/journal.pbio.0050242)
Supplement: Supplementary file 3 [file pbio.0050242.st003.doc]

**Supporting Table S3**

Results of the ASPEX sib_TDT for SNPs in the region of *COL29A1* for allergic sensitization in the discovery cohort.

| Locus | Position on | Location  in | MAF^a^ | Major/minor allele |  | All transmissions | | | |  | Paternal | | | |  | Maternal | | | |
| --- | --- | --- | --- | --- | --- | --- | --- | --- | --- | --- | --- | --- | --- | --- | --- | --- | --- | --- | --- |
|  | chromosome 3 | COL29A1 |  |  |  | Tb | NTc | χ2 | P value |  | T | NT | χ2 | P value |  | T | NT | χ2 | P value |
| rs1349747 | 131,543,410 | 5’ | 18.0 | **A**/G |  | 91 | 75 | 1.54 | 0.27 |  | 34 | 35 | 0.01 | 0.90 |  | 52 | 35 | 3.32 | 0.12 |
| rs1869319 | 131,547,433 | exon 1 | 9.4 | **C**/T |  | 53 | 48 | 0.25 | 0.73 |  | 19 | 24 | 0.33 | 0.67 |  | 31 | 21 | 1.53 | 0.33 |
| rs13095825 | 131,549,247 | intron 1 | 36.6 | **T**/C |  | 164 | 122 | 6.17 | 0.02 |  | 58 | 42 | 2.56 | 0.081 |  | 78 | 52 | 5.2 | 0.036 |
| rs1453243 | 131,575,875 | intron 2 | 30.5 | **G**/A |  | 141 | 107 | 4.66 | 0.048 |  | 51 | 36 | 2.59 | 0.1 |  | 67 | 48 | 3.14 | 0.11 |
| rs5852593 | 131,580,335 | intron 3 | 24.3 | **del**/T |  | 117 | 78 | 7.8 | 0.0085 |  | 55 | 31 | 6.7 | 0.02 |  | 48 | 33 | 2.78 | 0.095 |
| rs16845861 | 131,581,039 | exon 4 | 24.4 | **G**/A |  | 123 | 86 | 6.55 | 0.017 |  | 52 | 33 | 4.25 | 0.052 |  | 57 | 39 | 3.38 | 0.081 |
| rs1453242 | 131,586,229 | intron 4 | 24.3 | **C**/T |  | 126 | 86 | 7.55 | 0.011 |  | 58 | 35 | 5.69 | 0.027 |  | 56 | 39 | 3.04 | 0.095 |
| rs10212372 | 131,586,896 | exon 5 | 24.4 | **A**/G |  | 122 | 86 | 6.23 | 0.02 |  | 52 | 34 | 3.77 | 0.065 |  | 56 | 38 | 3.45 | 0.078 |
| A36603217 | 131,590,753 | intron 6 | 22.6 | **A**/G |  | 120 | 87 | 5.26 | 0.031 |  | 56 | 44 | 1.44 | 0.27 |  | 53 | 32 | 5.19 | 0.026 |
| rs10934938 | 131,596,325 | intron 7 | 22.0 | **T**/G |  | 118 | 82 | 6.48 | 0.017 |  | 57 | 41 | 2.61 | 0.12 |  | 52 | 32 | 4.76 | 0.031 |
| rs4688761 | 131,607,679 | exon 16 | 28.6 | **T**/C |  | 146 | 91 | 12.76 | 0.00072 |  | 62 | 38 | 5.76 | 0.018 |  | 58 | 27 | 11.31 | 0.0013 |
| A36624476 | 131,612,012 | exon 20 | 13.5 | **G**/C |  | 79 | 65 | 1.36 | 0.29 |  | 36 | 33 | 0.13 | 0.65 |  | 35 | 24 | 2.05 | 0.16 |
| rs1497309 | 131,616,310 | intron 22 | 28.7 | **C**/T |  | 144 | 98 | 8.74 | 0.0052 |  | 60 | 40 | 4.0 | 0.045 |  | 58 | 32 | 7.51 | 0.011 |
| rs7628563 | 131,617,253 | intron 23 | 15.1 | **A**/G |  | 96 | 63 | 6.85 | 0.017 |  | 46 | 25 | 6.21 | 0.01 |  | 37 | 25 | 2.32 | 0.16 |
| A36637742 | 131.625.270 | intron 28 | 27,8 | **del**/T |  | 141 | 104 | 5.59 | 0.028 |  | 61 | 43 | 3.12 | 0.09 |  | 55 | 36 | 3.97 | 0.063 |
| rs1566136 | 131,634,657 | intron 33 | 28.4 | **A**/G |  | 140 | 112 | 3.11 | 0.096 |  | 66 | 49 | 2.51 | 0.11 |  | 53 | 42 | 1.27 | 0.24 |
| rs9883988 | 131,645,085 | exon 36 | 6.3 | **G**/A |  | 44 | 26 | 4.63 | 0.057 |  | 26 | 15 | 2.95 | 0.10 |  | 15 | 8 | 2.13 | 0.23 |
| rs322113 | 131,670,507 | exon 38 | 7.8 | **C**/T |  | 29 | 26 | 0.16 | 0.8 |  | 16 | 13 | 0.31 | 0.77 |  | 13 | 13 | 0 | 1.0 |
| rs322115 | 131,672,716 | intron 39 | 18.2 | **A**/T |  | 80 | 64 | 1.78 | 0.26 |  | 37 | 30 | 0.73 | 0.43 |  | 36 | 27 | 1.29 | 0.36 |
| rs819091 | 131,678,260 | intron 40 | 23.7 | **G**/A |  | 112 | 84 | 4.0 | 0.062 |  | 52 | 41 | 1.3 | 0.31 |  | 47 | 30 | 3.75 | 0.061 |
| rs7624712 | 131,686,432 | 3’ | 11.6 | **A**/G |  | 48 | 47 | 0.01 | 1 |  | 24 | 22 | 0.09 | 0.66 |  | 22 | 23 | 0.02 | 0.80 |

^a^ MAF minor allele frequency

^b^ T number of transmissions

^c^ NT number of non-transmissions
