# Supplementary material for: Variants in a Novel Epidermal Collagen Gene (COL29A1) Are Associated with Atopic Dermatitis
Source: PLoS Biol. 2007 Sep 11;5(9):e242. doi: 10.1371/journal.pbio.0050242 (PMC1971127; doi:10.1371/journal.pbio.0050242)
Supplement: Supplementary file 4 [file pbio.0050242.st004.doc]

**Table S4**. Association analysis of single markers for allergic sensitization in the replication data set.

| Locus | Major/ |  | All transmissions | | | |  | Paternal | | | |  | Maternal | | | |
| --- | --- | --- | --- | --- | --- | --- | --- | --- | --- | --- | --- | --- | --- | --- | --- | --- |
|  | minor allele |  | T^b^ | NT^c^ | χ2 | P value^a^ |  | T | NT | χ2 | P value |  | T | NT | χ2 | P value |
| rs13095825 | T/C |  | 159 | 122 | 4.87 | 0.03 |  | 57 | 46 | 1.17 | 0.28 |  | 67 | 41 | 6.26 | 0.0073 |
| rs16845861 | G/A |  | 128 | 89 | 7.01 | 0,0072* |  | 54 | 36 | 3.6 | 0.05 |  | 55 | 34 | 4.96 | 0.02 |
| rs10212372 | A/G |  | 127 | 89 | 6.69 | 0.0097* |  | 54 | 36 | 3.6 | 0.05 |  | 54 | 34 | 4.55 | 0.024 |
| A36603217 | A/G |  | 122 | 81 | 8.28 | 0,0044* |  | 62 | 39 | 5.24 | 0.024 |  | 45 | 27 | 4.5 | 0.02 |
| rs10934938 | T/G |  | 123 | 83 | 7.77 | 0,0056* |  | 63 | 40 | 5.14 | 0.023 |  | 45 | 28 | 3.96 | 0.026 |
| rs4688761 | T/C |  | 138 | 96 | 7.54 | 0,0065* |  | 73 | 48 | 5.17 | 0.027 |  | 49 | 32 | 3.57 | 0.055 |
| A36637742 | del/T |  | 170 | 115 | 10.61 | 0,0010* |  | 74 | 53 | 3.47 | 0.07 |  | 71 | 37 | 10.7 | 0.00072* |
| rs9883988 | G/A |  | 56 | 31 | 7.18 | 0,0073* |  | 32 | 17 | 4.59 | 0.035 |  | 23 | 13 | 2.78 | 0.072 |

^a^ *P* values of the ASPEX sib TDT for allergic sensitization for all, paternal, and maternal transmissions. None of the paternal transmissions were significant after simulation. ** P* values significant after correction for multiple testing.
