# Supplementary material for: Variants in a Novel Epidermal Collagen Gene (COL29A1) Are Associated with Atopic Dermatitis
Source: PLoS Biol. 2007 Sep 11;5(9):e242. doi: 10.1371/journal.pbio.0050242 (PMC1971127; doi:10.1371/journal.pbio.0050242)
Supplement: Supplementary file 5 [file pbio.0050242.st005.doc]

**Table S5**. Haplotype analysis for allergic sensitization in the replication data set.

|  |  |  |  |  |  |  |  |  | Allergic sensitization | | | | | | |
| --- | --- | --- | --- | --- | --- | --- | --- | --- | --- | --- | --- | --- | --- | --- | --- |
| rs13095825 | rs16845861 | *rs*10212372 | A36603217 | *rs*10934938 | *rs*4688761 | A3667742 | rs9883988 |  | freq. | fam. | S | E(S) | Var(S) | Z | *P* value |
| 2 | 1 | 1 | 1 | 2 | . |  | . |  | 0.62 | 112 | 360.0 | 332.7 | 76.8 | 3.12 | 0.0018* |
| . | 1 | 1 | 1 | 2 | 1 |  | . |  | 0.68 | 103 | 352.7 | 324.3 | 69.2 | 3.42 | 0.00062* |
|  |  | 1 | 1 | 2 | 1 | 1 |  |  | 0.66 | 100 | 350.7 | 320.2 | 67.7 | 3.70 | 0.00021* |
| . | . |  | 1 | 2 | 1 | 1 | 2 |  | 0.70 | 96 | 313.9 | 313.9 | 70.5 | 3.9 | 0.000085* |

fbat statistic (Z) for 5-marker haplotype transmissions in the replication set adjusted for the correlation among siblings, reporting haplotype frequency (freq.) the number of informative families (fam.), the distribution (S), the expected distribution (E(S)), and the variance (Var(S)). * *P* values significant after correction for multiple testing.
